# Genome-Wide Screening Reveals an EMT Molecular Network Mediated by Sonic Hedgehog-Gli1 Signaling in Pancreatic Cancer Cells

**DOI:** 10.1371/journal.pone.0043119

**Published:** 2012-08-10

**Authors:** Xuanfu Xu, Yingqun Zhou, Chuangao Xie, Shu-mei Wei, Huizhong Gan, Shengli He, Fan Wang, Ling Xu, Jie Lu, Weiqi Dai, Lei He, Ping Chen, Xingpeng Wang, Chuanyong Guo

**Affiliations:** 1 Department of Gastroenterology, The Tenth People's Hospital of Shanghai, Tongji University, Shanghai, China; 2 Department of Gastroenterology, The Second Hospital of Zhejiang University, Hangzhou, Zhejiang Province, China; 3 Department of Gastroenterology, The First People's Hospital of Hefei, Anhui Medical University, Heifei, Anhui Province, China; 4 Department of Integrative Oncology, Minhang Branch of Fudan University Shanghai Cancer Center, Shanghai, China; Wayne State University School of Medicine, United States of America

## Abstract

**Aims:**

The role of sonic hedgehog (SHH) in epithelial mesenchymal transition (EMT) of pancreatic cancer (PC) is known, however, its mechanism is unclear. Because SHH promotes tumor development predominantly through Gli1, we sought to understand its mechanism by identifying Gli1 targets in pancreatic cancer cells.

**Methods:**

First, we investigated invasion, migration, and EMT in PC cells transfected with lentiviral Gli1 interference vectors or SHH over-expression vectors *in vitro* and *in vivo*. Next, we determined the target gene profiles of Gli1 in PC cells using cDNA microarray assays. Finally, the primary regulatory networks downstream of SHH-Gli1 signaling in PC cells were studied through functional analyses of these targets.

**Results:**

Our results indicate there is decreased E-cadherin expression upon increased expression of SHH/Gli1. Migration of PC cells increased significantly in a dose-dependent manner within 24 hours of Gli1 expression (*P*<0.05). The ratio of liver metastasis and intrasplenic miniature metastasis increased markedly upon activation of SHH-Gli1 signals in nude mice. Using cDNA microarray, we identified 278 upregulated and 59 downregulated genes upon Gli1 expression in AsPC-1 cells. The data indicate that SHH-Gli1 signals promote EMT by mediating a complex signaling network including TGFβ, Ras, Wnt, growth factors, PI3K/AKT, integrins, transmembrane 4 superfamily (TM4SF), and S100A4.

**Conclusion:**

Our results suggest that targeting the molecular connections established between SHH-Gli1 signaling and EMT could provide effective therapies for PC.

## Introduction

Sonic hedgehog (SHH) is involved in embryonic organogenesis as a morphogen. Inappropriate activation of SHH signals during pancreas formation results in agenesis and several pancreatic diseases [1]. SHH is excluded from the developing pancreas as well as the mature organ, but is upregulated in chronic pancreatitis, early pancreatic intraepithelial neoplasia (PanIN) lesions, and invasive pancreatic cancer (PC) [2]. Aberrant SHH upregulation was reported in subtotal human PC cells and might be a primary critical mediator of PC development [3].

The Hedgehog (HH) signaling pathway is closely related to tumor metastasis and prognosis in clinical studies and is required for PC tumor metastasis in orthotopic mouse models [3], [4]. Recently, this pathway was thought to orchestrate the reprogramming of cancer cells via epithelial mesenchymal transition (EMT). Interestingly, recent evidence found that SHH was significantly upregulated in gemcitabine-resistant PC cells that simultaneously express cancer stem cell (CSCs) markers [5]. Because the SHH-induced target gene products could contribute to the self-renewal, survival, and migration of cancer progenitor cells and Gli1 may play a crucial role in the malignant behavior of PC cells [6], [7], identifying Gli1 targets is a logical step to understand its mechanism in PC cells.

**Table 1:** The primer sequences for real time RT-PCR assays.

Gene	Primer Sequences	Annealing Temperature (°C)	Size (bp)
Gli1	F: 5′-TCTGCCCCCATTGCCCAC TTG-3′	56	480
	R: 5′-TACATAGCCCCCAGCCCATAC CTC-3′		
Shh	F: 5′-CGGAGCGAGGAAGGGA AAG-3′	56	262
	R: 5′-TTGGGGATAAACTGCTTGTA GGC-3′		
Patched1	5′-CGGCGTTCTCAATGGGCTGGT TTT-3′	54	376
	5′-GTGGGGCTGCTGTTTCGGGT TCG-3′		
GAPDH	F: 5′-ACGGATTTGGTCGTATT GGG-3′	54	208
	R: 5′-TGGAAGATGGTGATGGG ATT-3′		
E-cadherin	F: 5′- CAATGCCGCCATCGCT TAC -3′	56	421
	R: 5′- CAAAATGCCATCGTTGTTC ACT -3′		

The goal of this study was to provide a framework for the primary regulatory networks downstream of SHH-Gli1 signaling in PC cells. We also sought to determine if specific Gli1 target genes connect SHH-Gli1 signaling and EMT, thus providing a therapeutic strategy for PC.

## Materials and methods

### Cell culture

The PC cell lines (BxPC3, AsPC-1, and Panc-1 were all saved by the Chinese Academy of Sciences.) were cultured in RPMI-1640 supplemented with 10% fetal calf serum (FCS). All cells were incubated at 37°C in a humidified atmosphere of 5% CO_2_ in air.

### Vector construction and cell infection

Lentiviral transfer vectors for human Gli1 shRNA or SHH cDNA were constructed by Genechem Co., Ltd, Shanghai, China. This system includes the lentiviral vector pLVTHM, the envelope plasmid pMD2G, and the packaging plasmids pRsv-REV and pMDlg-pRRE. The lentivirus-SHH (L-SHH) contains a 3.3-kb SHH coding sequence and the lentivirus-Gli1i (L-Gli1i) contains small hairpin Gli1 RNA to the targeting sequence of the shRNA, as previously described (5′-CTCCACAGGCATACAGGAT-3′) [8]. The lentivirus-control (L-C) did not include Gli1 interference sequences or SHH cDNA sequences and served as control. Lentiviral constructs were verified by DNA sequencing. Recombinant lentivirus was produced by transiently transfecting 293T cells following a standard protocol. When BxPC3, AsPC-1, and Panc-1 cells were approximately 50% confluent (in RPMI-1640 containing 2% FCS), they were infected with the lentiviral constructs at MOI of 5. Cells were harvested after 72 hours for further experiments. To identify functional L-SHH and L-Gli1i constructs, we routinely analyzed SHH and Gli1 expression by qRT-PCR.

**Figure 1:**
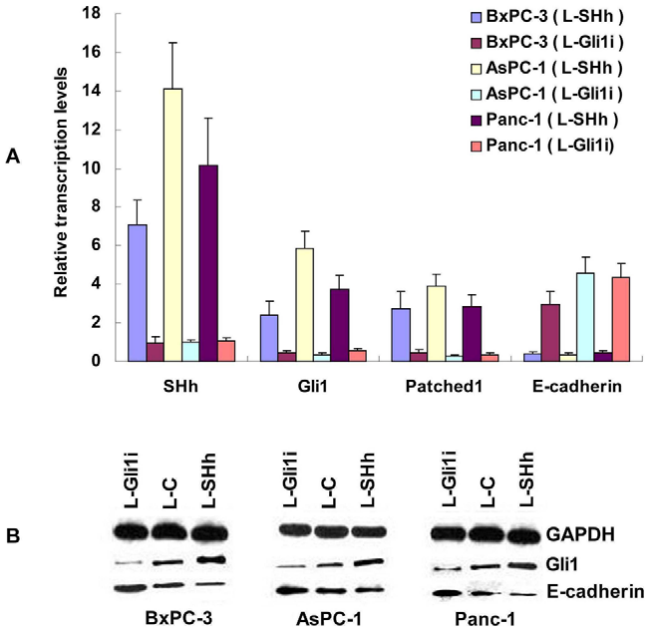
Lentiviral-Gli1i and -SHH transduction efficiency and PC cell EMT is regulated by SHH-Gli1 signaling. A: Expression of SHH, Gli1, Patched1, and E-cadherin mRNAs in the presence of L-Gli1i and SHH transduction. B: Western blot showing protein expression of Gli1, E-cadherin, and GAPDH in pancreatic cancer cell lines.

### RNA extraction and real time RT-PCR assays

Total RNA was extracted with Trizol reagent (Invitrogen Corporation, Carlsbad, CA, USA) according to the manufacturer's protocol. Total RNA (100 ng) was reverse transcribed in 20 μl volume and 2 μl cDNA was used for PCR, according to the manufacturer's instructions. (TaKaRa Biotechnology, Dalian, China). The primer sequences are shown in Table 1. CT (cycle threshold) values were standardized to CT values of GAPDH.

**Figure 2:**
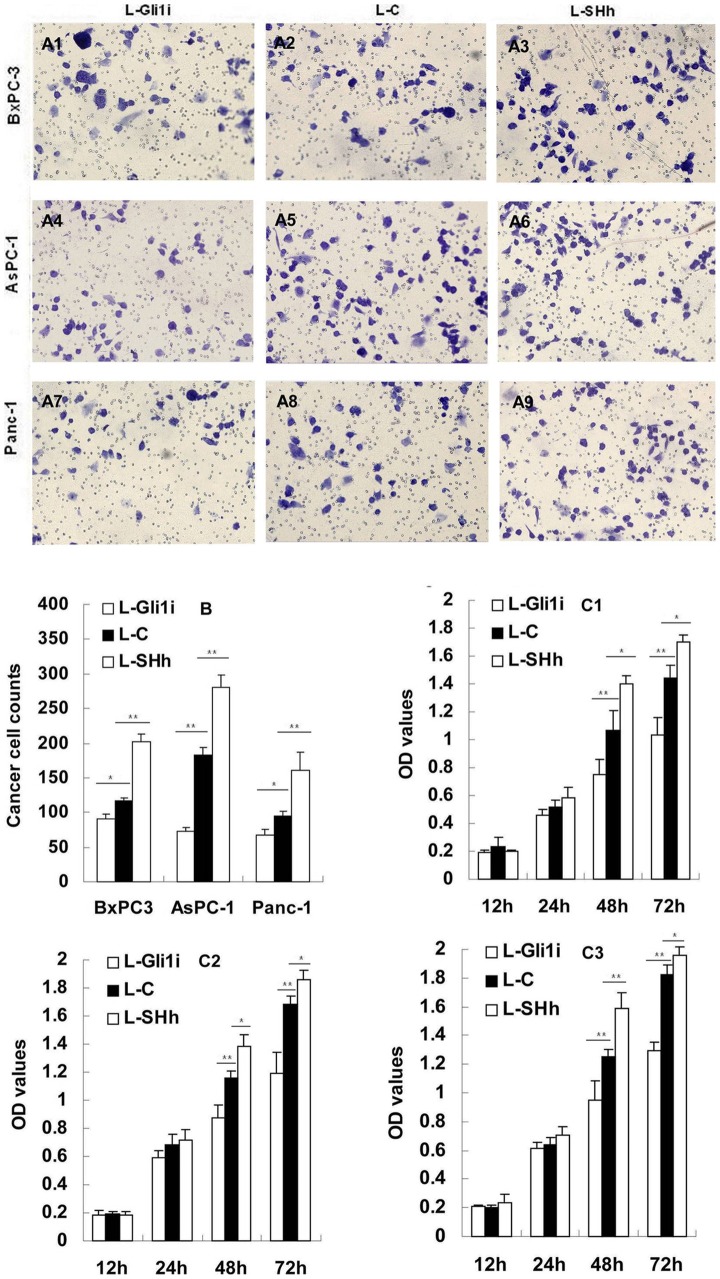
SHH-Gli1 signaling regulates PC cell invasion and migration. A1-9: Crystal violet staining of PC cells through polycarbonate membrane pores (×200 magnification). B: Cell counts of migrating PC cells as analyzed by transwell assay. C1-3: Cell proliferation as determined by MTT (C1: BxPC-3 cells; C2: AsPC-1 cells; C3: Panc-1 cells). *P<0.05, **P<0.01.

### Protein extraction and western blotting assays

Total protein was extracted with RIPA buffer according to standard methods and samples were normalized for protein content using a commercially available kit (Bio-Rad Laboratories Inc Philadelphia, PA USA). Protein samples were separated by 6% SDS-PAGE (for Gli1 protein) and 12% SDS-PAGE (for SHH, E-cadherin, and GAPDH). Proteins were transferred to PVDF membranes and membranes were incubated for 2 h in TBST buffer, followed by incubation overnight at 4°C with the primary antibodies [1∶1000 (v/v) for SHH, E-cadherin, or GAPDH and 1∶500 (v/v) for Gli1] in blocking solution and visualization using the ECL detection system (GE Healthcare Biosciences, Piscataway, NJ, USA).

**Figure 3:**
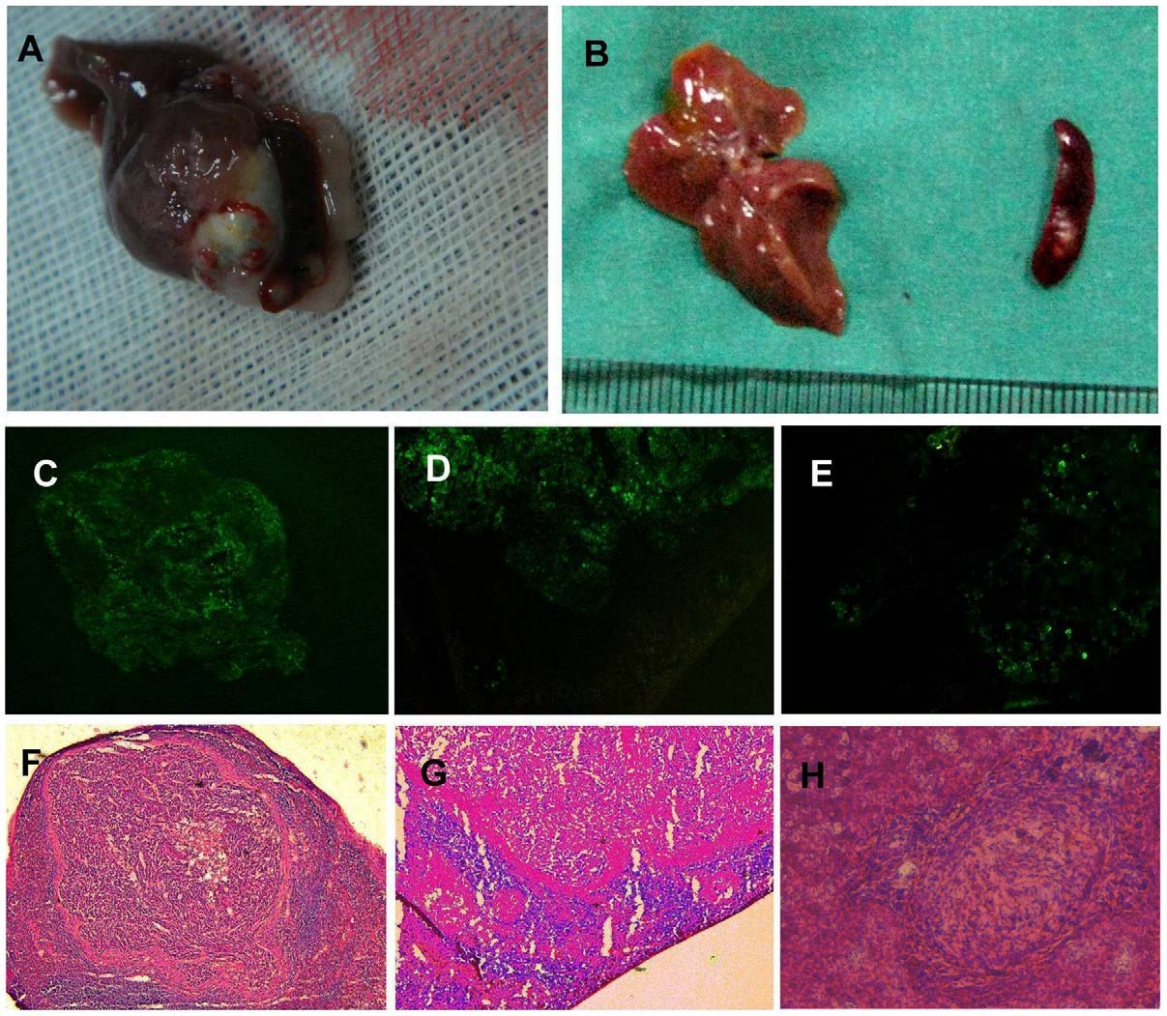
Experimental metastasis model of intrasplenic inoculation into nude mice. A: Spleen tumors and liver metastases in a macroscopic specimen of the L-SHH group. B: Spleen tumors and liver metastases in a macroscopic specimen of the L-C group. C, D, and E: Fluorescence microscopy images. F, G, and H: Lightmicroscopy images. (C, F: Spleen tumors from the L-Gli1i group; D, G: Intrasplenic miniature metastases from the L-C group; E, H: Liver metastases from the L-SHH group). *P<0.05, **P<0.01.

**Table 2:** Intrasplenic and liver metastases induced by splenic injection in nude mice.

Groups	Tumorigenicity	Metastases	Liver metastasis	
			Incidence	Number
L-Gli1i	8(10)	2.6	3(8)	2.7
L-C	8(9)	4.9	5(8)	4.2
L-SHH	9(9)	8.9	9(9)	6.7

### Transwell assays

Cell invasion assays (24-well sample kits; Chemicon, Bedford, MA, USA) were used to study PC cell line invasion and migration. Briefly, PC cells (1×10^5^) were separately seeded in serum-free media in Matrigel pre-coated transwell chambers (upper chamber), which contained polycarbonate membranes with 8-μm pores. Media containing 2% FCS was added into the bottom chamber. The transwell chambers were then placed on the 24-well plates. After incubation for 24 h, migration of PC cells was determined by photographing the membrane through the microscope. Counts were recorded from the 5 areas with the highest cell concentrations at high power magnification (×200). The mean value of the fields was considered the migration count of PC cells.

**Figure 4:**
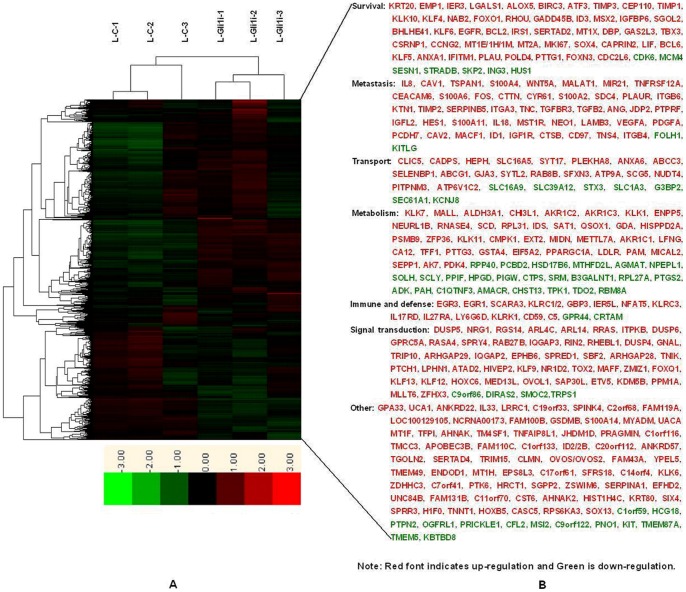
cDNA microarray analyses of Gli1 target genes in AsPC-1 cells. A: cDNA microarray data cluster comparing L-C and L-Gli1i cells. B: Functional classification of differentially expressed genes. See also Table 2.

**Table 3:** The target genes upon Gli1 in AsPC-1 cells.

Public ID	Gene Symbol	Gene Title	Fold
AI732381	KRT20	keratin 20	7.74061
NM_005046	KLK7	kallikrein-related peptidase 7	6.69379
NM_001423	EMP1	epithelial membrane protein 1	5.70263
NM_004430	EGR3	early growth response 3	5.49528
U16996	DUSP5	dual specificity phosphatase 5	4.74988
NM_005814	GPA33	glycoprotein A33 (transmembrane)	4.53642
AA702248	UCA1	urothelial cancer associated 1	4.4864
NM_000584	IL8	interleukin 8	4.14464
NM_001323	CST6	cystatin E/M	4.04605
NM_003897	IER3	immediate early response 3	4.01373
AU147399	CAV1	caveolin 1, caveolae protein, 22 kDa	3.9368
NM_002305	LGALS1	lectin, galactoside-binding, soluble, 1	3.52748
AF133425	TSPAN1	tetraspanin 1	3.42412
NM_002961	S100A4	S100 calcium binding protein A4	3.42195
L12260	NRG1	neuregulin 1	3.38084
AL049313	CLIC5	chloride intracellular channel 5	3.19394
NM_003392	WNT5A	wingless-type MMTV integration site family, member 5A	3.19181
W80468	MALAT1	metastasis associated lung adenocarcinoma transcript 1 (non-protein coding)	3.1871
AI925518	ANKRD22	ankyrin repeat domain 22	3.16604
BC003179	MALL	mal, T-cell differentiation protein-like	3.11744
AI935123	AHNAK2	AHNAK nucleoprotein 2	3.09801
AF037195	RGS14	regulator of G-protein signaling 14	3.09657
NM_000698	ALOX5	arachidonate 5-lipoxygenase	3.02439
AV733950	EGR1	early growth response 1	2.95766
BF674052	MIR21	microRNA 21	2.94012
NM_000691	ALDH3A1	aldehyde dehydrogenase 3 family, memberA1	2.93342
NM_016639	TNFRSF12A	tumor necrosis factor receptor superfamily, member 12A	2.93325
M18728	CEACAM6	carcinoembryonic antigen-related cell adhesion molecule 6	2.86749
NM_014624	S100A6	S100 calcium binding protein A6	2.83904
AI912173	CADPS	Ca++-dependent secretion activator	2.81662
BC004490	FOS	v-fos FBJ murine osteosarcoma viral oncogene homolog	2.7865
AB024518	IL33	interleukin 33	2.70788
U37546	BIRC3	baculoviral IAP repeat-containing 3	2.6776
M80927	CHI3L1	chitinase 3-like 1 (cartilage glycoprotein-39)	2.67293
NM_003542	HIST1H4C	histone cluster 1, H4c	2.65858
AI139629	ATAD2	ATPase family, AAA domain containing 2	2.6584
NM_001674	ATF3	activating transcription factor 3	2.64602
AB007830	SCARA3	scavenger receptor class A, member 3	2.63993
BG475299	CTTN	cortactin	2.63957
NM_001554	CYR61	cysteine-rich, angiogenic inducer, 61	2.61643
NM_000362	TIMP3	TIMP metallopeptidase inhibitor 3	2.57069
NM_005978	S100A2	S100 calcium binding protein A2	2.54785
M33376	AKR1C2	aldo-keto reductase family 1, member C2	2.53992
AB018580	AKR1C3	aldo-keto reductase family 1, member C3	2.50169
NM_002999	SDC4	syndecan 4	2.47093
BG435404	ARL4C	ADP-ribosylation factor-like 4C	2.4621
AL023584	HIVEP2	human immunodeficiency virus type I enhancer binding protein 2	2.43299
U08839	PLAUR	plasminogen activator, urokinase receptor	2.42304
NM_014799	HEPH	hephaestin	2.39676
AL162069	KRT80	keratin 80	2.39224
AK026736	ITGB6	integrin, beta 6	2.37268
AI554514	SIX4	SIX homeobox 4	2.34565
NM_007018	CEP110	centrosomal protein 110 kDa	2.32987
NM_001206	KLF9	Kruppel-like factor 9	2.3297
NM_025168	LRRC1	leucine rich repeat containing 1	2.32702
BF589024	KTN1	kinectin 1 (kinesin receptor)	2.32234
NM_003254	TIMP1	TIMP metallopeptidase inhibitor 1	2.3168
BF107565	TIMP2	TIMP metallopeptidase inhibitor 2	2.31548
AF213678	C19orf33	chromosome 19 open reading frame 33	2.31041
NM_005416	SPRR3	small proline-rich protein 3	2.31004
NM_004695	SLC16A5	similar to MCT///solute carrier family 16, member 5	2.30392
NM_025047	ARL14	ADP-ribosylation factor-like 14	2.28222
AI582818	SYT17	Synaptotagmin XVII	2.27965
NM_002639	SERPINB5	serpin peptidase inhibitor, clade B (ovalbumin), member 5	2.25488
L10038	KLK1	kallikrein 1	2.25193
NM_014471	SPINK4	serine peptidase inhibitor, Kazal type 4	2.25172
NM_002204	ITGA3	integrin, alpha 3	2.25128
NM_006270	RRAS	related RAS viral (r-ras) oncogene homolog	2.24532
AI761621	NR1D2	nuclear receptor subfamily 1, group D, member 2	2.23073
AA211909	TOX2	TOX high mobility group box family member 2	2.23018
BC002710	KLK10	kallikrein-related peptidase 10	2.21885
AU147777	C2orf68	chromosome 2 open reading frame 68	2.21694
NM_002160	TNC	tenascin C	2.20758
AW193698	TGFBR3	transforming growth factor, beta receptor III	2.20145
NM_145280	FAM119A	family with sequence similarity 119, member A	2.19395
AA609053	ENPP5	ectonucleotide pyrophosphatase/phosphodiesterase 5 (putative function)	2.19289
AU145950	TGFB2	transforming growth factor, beta 2	2.18613
AW471181	LOC100129105	similar to hCG1821214	2.17711
AK026748	NEURL1B	neuralized homolog 1B (Drosophila)	2.17305
BF514079	KLF4	Kruppel-like factor 4 (gut)	2.16858
NM_002260	KLRC1/2	killer cell lectin-like receptor subfamily C, member ½	2.16641
NM_002221	ITPKB	inositol 1,4,5-trisphosphate 3-kinase B	2.16
NM_001145	ANG	angiogenin, ribonuclease, RNase A family, 5	2.15958
AI761728	RNASE4	ribonuclease, RNase A family, 4	2.14588
AI821565	NCRNA00173	non-protein coding RNA 173	2.14337
BC005047	DUSP6	dual specificity phosphatase 6	2.13885
AB032261	SCD	stearoyl-CoA desaturase (delta-9-desaturase)	2.12989
AI024869	FAM100B	family with sequence similarity 100, member B	2.12696
H98994	PLEKHA8	Pleckstrin homology domain containing, family A (phosphoinositide binding specific) member 8	2.12631
AA716425	JDP2	Jun dimerization protein 2	2.12282
NM_003979	GPRC5A	G protein-coupled receptor, family C, group 5, member A	2.12099
AL136680	GBP3	guanylate binding protein 3	2.11632
BF337329	NAB2	NGFI-A binding protein 2 (EGR1 binding protein 2)	2.10899
AL021977	MAFF	v-maf musculoaponeurotic fibrosarcoma oncogene homolog F (avian)	2.10067
NM_018530	GSDMB	gasdermin B	2.10044
NM_020672	S100A14	S100 calcium binding protein A14	2.09621
AI348010	RPL31	ribosomal protein L31	2.08777
BF110608	IER5L	immediate early response 5-like	2.08615
AW117498	FOXO1	forkhead box O1	2.08564
AF070622	ZMIZ1	zinc finger, MIZ-type containing 1	2.08135
AV703259	IDS	iduronate 2-sulfatase	2.08022
BE971383	SAT1	spermidine/spermine N1-acetyltransferase 1	2.07809
BE908995	MYADM	myeloid-associated differentiation marker	2.0759
AL096776	RHOU	ras homolog gene family, member U	2.073
BC000145	H1F0	H1 histone family, member 0	2.0689
NM_015675	GADD45B	growth arrest and DNA-damage-inducible, beta	2.05986
NM_001155	ANXA6	annexin A6	2.05797
BE301252	QSOX1	quiescin Q6 sulfhydryl oxidase 1	2.05632
NM_020037	ABCC3	ATP-binding cassette, sub-family C (CFTR/MRP), member 3	2.0476
NM_002167	ID3	inhibitor of DNA binding 3, dominant negative helix-loop-helix protein	2.04538
AJ005683	NFAT5	nuclear factor of activated T-cells 5, tonicity-responsive	2.03963
AF019638	GDA	guanine deaminase	2.03934
AB011110	RASA4	RAS p21 protein activator 4	2.0382
D31771	MSX2	msh homeobox 2	2.03765
NM_002178	IGFBP6	insulin-like growth factor binding protein 6	2.0353
NM_002840	PTPRF	protein tyrosine phosphatase, receptor type, F	2.02798
W48843	SPRY4	sprouty homolog 4 (Drosophila)	2.02513
AF322916	UACA	uveal autoantigen with coiled-coil domains and ankyrin repeats	2.01803
AF543190	HISPPD2A	histidine acid phosphatase domain containing 2A	2.01272
NM_003944	SELENBP1	selenium binding protein 1	2.00877
AI806131	IGFL2	IGF-like family member 2	2.00535
NM_002800	PSMB9	proteasome (prosome, macropain) subunit, beta type, 9	2.00438
AJ011712	TNNT1	troponin T type 1 (skeletal, slow)	2.00126
AW965339	SGOL2	shugoshin-like 2 (S. pombe)	1.99085
BE973687	HES1	hairy and enhancer of split 1, (Drosophila)	1.98785
NM_003407	ZFP36	zinc finger protein 36, C3H type, homolog (mouse)	1.98751
NM_006853	KLK11	kallikrein-related peptidase 11	1.98605
BF246115	MT1F	metallothionein 1F	1.98245
AI676059	FOXQ1	forkhead box Q1	1.98014
AF021834	TFPI	tissue factor pathway inhibitor (lipoprotein-associated coagulation inhibitor)	1.97581
AL390127	KLF13	Kruppel-like factor 13	1.97416
NM_016308	CMPK1	cytidine monophosphate (UMP-CMP) kinase 1, cytosolic	1.97344
NM_000401	EXT2	exostoses (multiple) 2	1.96824
BG287862	AHNAK	AHNAK nucleoprotein	1.9654
NM_002261	KLRC3	killer cell lectin-like receptor subfamily C, member 3	1.96114
AI189753	TM4SF1	transmembrane 4 L six family member 1	1.95878
BF438386	RAB27B	RAB27B, member RAS oncogene family	1.95437
AA020010	KLF12	Kruppel-like factor 12	1.95402
AL512725	MIDN	Midnolin	1.94948
BE857425	BHLHE41	basic helix-loop-helix family, member e41	1.94834
BU683415	KLF6	Kruppel-like factor 6	1.94815
BF338045	TNFAIP8L1	tumor necrosis factor, alpha-induced protein 8-like 1	1.94402
NM_014033	METTL7A	methyltransferase like 7A	1.93619
S68290	AKR1C1	aldo-keto reductase family 1, member C1	1.93607
NM_004503	HOXC6	homeobox C6	1.93452
BE217882	JHDM1D	jumonji C domain containing histone demethylase 1 homolog D (S. cerevisiae)	1.93221
BF739767	PRAGMIN	homolog of rat pragma of Rnd2	1.93063
AW007080	IL17RD	interleukin 17 receptor D	1.92254
NM_021039	S100A11	S100 calcium binding protein A11	1.92157
AI983115	IL27RA	interleukin 27 receptor, alpha	1.92132
NM_002147	HOXB5	homeobox B5	1.91937
NM_021246	LY6G6D	lymphocyte antigen 6 complex, locus G6D	1.91452
NM_001562	IL18	interleukin 18 (interferon-gamma-inducing factor)	1.91426
NM_002447	MST1R	macrophage stimulating 1 receptor (c-met-related tyrosine kinase)	1.9139
AL355708	NEO1	neogenin homolog 1 (chicken)	1.90936
NM_024115	C1orf116	chromosome 1 open reading frame 116	1.90905
AF439512	KLRK1	killer cell lectin-like receptor subfamily K, member 1	1.90656
AW157070	EGFR	epidermal growth factor receptor	1.90531
AW151924	LFNG	LFNG O-fucosylpeptide 3-beta-N-acetylglucosaminyltransferase	1.90039
AA133277	BCL2	BCL2	1.90004
AI474666	IRS1	insulin receptor substrate 1	1.8898
BF752277	CA12	carbonic anhydrase XII	1.884
NM_004915	ABCG1	ATP-binding cassette, sub-family G (WHITE), member 1	1.88313
N51717	TMCC3	transmembrane and coiled-coil domain family 3	1.88267
NM_014755	SERTAD2	SERTA domain containing 2	1.88025
NM_004900	APOBEC3B	apolipoprotein B mRNA editing enzyme, catalytic polypeptide-like 3B	1.87712
AL133033	MED13L	mediator complex subunit 13-like	1.87401
NM_005952	MT1X	metallothionein 1X	1.87389
L25541	LAMB3	laminin, beta 3	1.87302
AF022375	VEGFA	vascular endothelial growth factor A	1.87138
AA588400	OVOL1	ovo-like 1(Drosophila)	1.86924
BF726530	GJA3	gap junction protein, alpha 3, 46 kDa	1.86225
NM_144508	CASC5	cancer susceptibility candidate 5	1.85939
U79283	DBP	D site of albumin promoter (albumin D-box) binding protein	1.85936
AW271106	IQGAP3	IQ motif containing GTPase activating protein 3	1.85751
N21426	SYTL2	Synaptotagmin-like 2	1.85066
AI860012	GAS2L3	Growth arrest-specific 2 like 3	1.847
NM_002607	PDGFA	platelet-derived growth factor alpha polypeptide	1.83526
NM_003225	TFF1	trefoil factor 1	1.83446
AL136924	RIN2	Ras and Rab interactor 2	1.83375
AI674565	FAM110C	family with sequence similarity 110, member C	1.8328
BF508679	C1orf133	chromosome 1 open reading frame 133	1.83104
NM_021000	PTTG3	pituitary tumor-transforming 3	1.83022
AI819238	ID2	inhibitor of DNA binding 2, dominant negative helix-loop-helix protein	1.82891
NM_001512	GSTA4	glutathione S-transferase alpha 4	1.82147
BG498334	RPS6KA3	ribosomal protein S6 kinase, 90 kDa, polypeptide 3	1.82032
NM_016569	TBX3	T-box 3	1.81904
AL034550	C20orf112	chromosome 20 open reading frame 112	1.81828
BE669553	ANKRD57	ankyrin repeat domain 57	1.81439
AI862477	SAP30L	SAP30-like	1.81113
AI091372	CSRNP1	cysteine-serine-rich nuclear protein 1	1.8103
AI807023	RAB8B	RAB8B, member RAS oncogene family	1.80823
AW134535	CCNG2	cyclin G2	1.80731
BC028219	TGOLN2	trans-golgi network protein 2	1.8022
BC014155	RHEBL1	Ras homolog enriched in brain like 1	1.80161
NM_001394	DUSP4	dual specificity phosphatase 4	1.79908
AV747725	EIF5A2	eukaryotic translation initiation factor 5A2	1.79722
AU146709	SERTAD4	SERTA domain containing 4	1.79659
AF220133	TRIM15	tripartite motif-containing 15	1.79617
AL031602	MT1E/1H/1M	metallothionein 1E/1H/1M	1.79612
AI082827	GNAL	guanine nucleotide binding protein (G protein), alpha activating activity polypeptide, olfactory type	1.79573
NM_005953	MT2A	metallothionein 2A	1.79486
NM_024734	CLMN	calmin (calponin-like, transmembrane)	1.7933
AW594320	OVOS/OVOS2	similar to hCG38149///ovostatin///ovostatin 2	1.792
AF116571	SOX13	SRY (sex determining region Y)-box 13	1.79158
NM_013261	PPARGC1A	peroxisome proliferator-activated receptor gamma, coactivator 1 alpha	1.79113
NM_004240	TRIP10	thyroid hormone receptor interactor 10	1.79072
BE644809	PCDH7	protocadherin 7	1.78886
NM_004454	ETV5	ets variant 5	1.78653
BF001806	MKI67	antigen identified by monoclonal antibody Ki-67	1.78641
BG528420	SOX4	SRY (sex determining region Y)-box 4	1.78459
AW264102	FAM43A	family with sequence similarity 43, member A	1.78133
NM_023925	CAPRIN2	caprin family member 2	1.78062
NM_016061	YPEL5	yippee-like 5 (Drosophila)	1.77979
NM_002309	LIF	leukemia inhibitory factor	1.77963
AL541655	TMEM49	transmembrane protein 49	1.77918
AF131747	ENDOD1	endonuclease domain containing 1	1.77616
BF197655	CAV2	caveolin 2	1.77602
NM_000527	LDLR	low density lipoprotein receptor	1.77329
NM_030971	SFXN3	sideroflexin 3	1.7732
NM_004815	ARHGAP29	Rho GTPase activating protein 29	1.77236
NM_005951	MT1H	metallothionein 1H	1.77077
NM_024526	EPS8L3	EPS8-like 3	1.76872
NM_006633	IQGAP2	IQ motif containing GTPase activating protein 2	1.76851
AB014511	ATP9A	ATPase, class II, type 9A	1.76617
BF975929	C17orf61	chromosome 17 open reading frame 61	1.76436
NM_001706	BCL6	B-cell CLL/lymphoma 6	1.76343
BE379006	CD59	CD59 molecule, complement regulatory protein	1.76235
AW081113	SFRS18	splicing factor, arginine/serine-rich 18	1.76215
AI932310	C14orf4	chromosome 14 open reading frame 4	1.75504
AB029290	MACF1	microtubule-actin crosslinking factor 1	1.75486
NM_006618	KDM5B	lysine (K)-specific demethylase 5B	1.75327
NM_004445	EPHB6	EPH receptor B6	1.75236
BF342524	SPRED1	sprouty-related, EVH1 domain containing 1	1.75156
AB030824	KLF5	Kruppel-like factor 5 (intestinal)	1.75153
BF038548	PAM	peptidylglycine alpha-amidating monooxygenase	1.74921
D13889	ID1	inhibitor of DNA binding 1, dominant negative helix-loop-helix protein	1.74845
AW276572	SBF2	SET binding factor 2	1.74723
AI935647	ARHGAP28	Rho GTPase activating protein 28	1.7444
NM_002774	KLK6	kallikrein-related peptidase 6	1.74312
BE965029	MICAL2	microtubule associated monoxygenase, calponin and LIM domain containing 2	1.73804
NM_000700	ANXA1	annexin A1	1.73654
R59093	TNIK	TRAF2 and NCK interacting kinase	1.73504
BF111925	ZDHHC3	zinc finger, DHHC-type containing 3	1.73496
NM_003020	SCG5	secretogranin V (7B2 protein)	1.73476
W73230	C7orf41	chromosome 7 open reading frame 41	1.73475
NM_005410	SEPP1	selenoprotein P, plasma, 1	1.73357
AL044092	IGF1R	insulin-like growth factor 1 receptor	1.73199
AA749101	IFITM1	interferon induced transmembrane protein 1 (9–27)	1.73188
W47179	CTSB	cathepsin B	1.72986
NM_005975	PTK6	PTK6 protein tyrosine kinase 6	1.7249
AW511135	NUDT4	nudix (nucleoside diphosphate linked moiety X)-type motif 4	1.72406
NM_001784	CD97	CD97 molecule	1.72395
K03226	PLAU	plasminogen activator, urokinase	1.72296
NM_021173	POLD4	polymerase (DNA-directed), delta 4	1.7214
AA886888	PPM1A	protein phosphatase 1A (formerly 2C), magnesium-dependent, alpha isoform	1.72092
AA158731	TNS4	tensin 4	1.71933
NM_152327	AK7	adenylate kinase 7	1.7149
NM_004219	PTTG1	pituitary tumor-transforming 1	1.7148
AI521254	HRCT1	histidine rich carboxyl terminus 1	1.7145
AI800110	SGPP2	sphingosine-1-phosphate phosphotase 2	1.71439
AV707102	PDK4	pyruvate dehydrogenase kinase, isozyme 4	1.71397
BG054916	PTCH1	patched homolog 1 (Drosophila)	1.71341
AI692595	ZSWIM6	zinc finger, SWIM-type containing 6	1.71248
AF119873	SERPINA1	serpin peptidase inhibitor, clade A (alpha-1 antiproteinase, antitrypsin), member 1	1.71135
AW664179	EFHD2	EF-hand domain family, member D2	1.71043
NM_000213	ITGB4	integrin, beta 4	1.70999
NM_024679	LPHN1	latrophilin 1	1.70907
BF063164	PITPNM3	PITPNM family member 3	1.70843
NM_001735	C5	complement component 5	1.70584
AL021707	UNC84B	unc-84 homolog B (C. elegans)	1.70583
NM_014690	FAM131B	family with sequence similarity 131, member B	1.70547
BG024886	MLLT6	myeloid/lymphoid or mixed-lineage leukemia (trithorax homolog, Drosophila); translocated to, 6	1.70508
AW051527	FOXN3	forkhead box N3	1.70455
AB028951	CDC2L6	cell division cycle 2-like 6 (CDK8-like)	1.70418
BG402859	ZFHX3	zinc finger homeobox 3	1.70332
BC006128	C11orf70	chromosome 11 open reading frame 70	1.70168
NM_144583	ATP6V1C2	ATPase, H+ transporting, lysosomal 42 kDa, V1 subunit C2	1.70137
NM_006638	RPP40	ribonuclease P/MRP 40 kDa subunit	0.58782
AL136721	PCBD2	pterin-4 alpha-carbinolamine dehydratase/dimerization cofactor of hepatocyte nuclear factor 1 alpha (TCF1) 2	0.58533
U89281	HSD17B6	hydroxysteroid (17-beta) dehydrogenase 6 homolog (mouse)	0.58343
BF185922	MTHFD2L	methylenetetrahydrofolate dehydrogenase (NADP+ dependent) 2-like	0.58139
BE502436	C1orf59	chromosome 1 open reading frame 59	0.57847
AA430072	HCG18	HLA complex group 18	0.57488
NM_002828	PTPN2	protein tyrosine phosphatase, non-receptor type 2	0.57432
NM_024576	OGFRL1	opioid growth factor receptor-like 1	0.57366
NM_004778	GPR44	G protein-coupled receptor 44	0.56901
AA404269	PRICKLE1	prickle homolog 1 (Drosophila)	0.56846
BC005090	AGMAT	agmatine ureohydrolase (agmatinase)	0.56447
NM_024718	C9orf86	chromosome 9 open reading frame 86	0.56282
AV726166	CFL2	cofilin 2 (muscle)	0.56207
AW274756	CDK6	cyclin-dependent kinase 6	0.56119
BG401568	SLC16A9	solute carrier family 16, member 9 (monocarboxylic acid transporter 9)	0.56011
AL577823	NPEPL1	Aminopeptidase-like 1	0.55752
BF433759	SOLH	small optic lobes homolog (Drosophila)	0.55631
AA911739	SCLY	Selenocysteine lyase	0.55584
NM_005729	PPIF	peptidylprolyl isomerase F	0.55446
BE000929	MSI2	musashi homolog 2 (Drosophila)	0.55395
AF264784	TRPS1	trichorhinophalangeal syndrome I	0.55347
BC041970	C9orf122	chromosome 9 open reading frame 122	0.54891
AV733347	PNO1	partner of NOB1 homolog (S. cerevisiae)	0.5455
AL574184	HPGD	hydroxyprostaglandin dehydrogenase 15-(NAD)	0.54429
NM_152725	SLC39A12	solute carrier family 39 (zinc transporter), member 12	0.54364
AI936566	MCM4	minichromosome maintenance complex component 4	0.54165
BF037819	PIGW	phosphatidylinositol glycan anchor biosynthesis, class W	0.54099
AJ002077	STX3	syntaxin 3	0.53917
NM_000222	KIT	v-kit Hardy-Zuckerman 4 feline sarcoma viral oncogene homolog	0.52724
NM_014454	SESN1	sestrin 1	0.527
NM_001905	CTPS	CTP synthase	0.52427
NM_004172	SLC1A3	solute carrier family 1 (glial high affinity glutamate transporter), member 3	0.52231
NM_012297	G3BP2	GTPase activating protein (SH3 domain) binding protein 2	0.51989
AB038950	STRADB	STE20-related kinase adaptor beta	0.51532
AW168915	FOLH1	folate hydrolase (prostate-specific membrane antigen) 1	0.51296
NM_003132	SRM	spermidine synthase	0.51065
AB050855	B3GALNT1	beta-1,3-N-acetylgalactosaminyltransferase 1 (globoside blood group)	0.50955
AF346602	SEC61A1	Sec61 alpha 1 subunit (S. cerevisiae)	0.50743
BC005335	TMEM87A	transmembrane protein 87A	0.50511
BF224146	TMEM5	transmembrane protein 5	0.50445
BC001441	SKP2	S-phase kinase-associated protein 2 (p45)	0.49395
BC004284	RPL27A	ribosomal protein L27a	0.49291
NM_000963	PTGS2	prostaglandin-endoperoxide synthase 2	0.49211
NM_001123	ADK	adenosine kinase	0.48824
NM_019604	CRTAM	cytotoxic and regulatory T cell molecule	0.48563
NM_000277	PAH	phenylalanine hydroxylase	0.4723
AF161419	ING3	inhibitor of growth family, member 3	0.46565
NM_004507	HUS1	HUS1 checkpoint homolog (S. pombe)	0.46307
AA888589	C1QTNF3	C1q and tumor necrosis factor related protein 3	0.45976
BF514158	KCNJ8	potassium inwardly-rectifying channel, subfamily J, member 8	0.40579
NM_017594	DIRAS2	DIRAS family, GTP-binding RAS-like 2	0.40548
AA669114	KBTBD8	kelch repeat and BTB (POZ) domain containing 8	0.38891
AI796120	AMACR/C1QTNF3	alpha-methylacyl-CoA racemase///C1q and tumor necrosis factor related protein 3	0.38848
AA677272	CHST13	carbohydrate (chondroitin 4) sulfotransferase 13	0.37567
NM_022445	TPK1	thiamin pyrophosphokinase 1	0.37373
NM_005651	TDO2	tryptophan 2,3-dioxygenase	0.36276
AB014737	SMOC2	SPARC related modular calcium binding 2	0.34035
AF119835	KITLG	KIT ligand	0.32023
BC017770	RBM8A	RNA binding motif protein 8A	0.22761

**Table 4:** PC-related genes and Hedgehog-related genes reported previously in the target genes profile data.

Public ID	Gene Symbol	PC-related	Hedgehog-related
AA133277	BCL2	P	P*
NM_002178	IGFBP6	P	P*
BG054916	PTCH1	P	P*
NM_003392	WNT5A	P	P*
D31771	MSX2	P	P*
U37546	BIRC3	P	P
BF514079	KLF4	P	P
K03226	PLAU	P	P
AW157070	EGFR	P	P
BE973687	HES1	P	P
NM_000584	IL8	P	P
NM_001145	ANG	P	P
AF022375	VEGFA	P	P
NM_002961	S100A4	P	P
W47179	CTSB	P	P
NM_020037	ABCC3	P	
NM_004219	PTTG1	P	
AI732381	KRT20	P	
NM_002167	ID3	P	
AB030824	KLF5	P	
NM_000700	ANXA1	P	
BC002710	KLK10	P	
BU683415	KLF6	P	
BF001806	MKI67	P	
NM_000698	ALOX5	P	
NM_001323	CST6	P	
NM_006633	IQGAP2	P	
L12260	NRG1	P	
W48843	SPRY4	P	
AF021834	TFPI	P	
NM_002774	KLK6	P	
AF119873	SERPINA1	P	
AL044092	IGF1R	P	
U08839	PLAUR	P	
BF589024	KTN1	P	
NM_002447	MST1R	P	
AA158731	TNS4	P	
BF674052	MIR21	P	
L25541	LAMB3	P	
NM_001562	IL18	P	
NM_002607	PDGFA	P	
NM_005978	S100A2	P	
NM_002999	SDC4	P	
M18728	CEACAM6	P	
NM_014624	S100A6	P	
NM_001512	GSTA4	P	
NM_005046	KLK7	P	
M80927	CHI3L1	P	
BE301252	QSOX1	P	
S68290	AKR1C1	P	
NM_003225	TFF1	P	
BE379006	CD59	P	
NM_001554	CYR61	P	
NM_002204	ITGA3	P	
AU147399	CAV1	P	
NM_002160	TNC	P	
NM_021039	S100A11	P	
BC004490	FOS	P	
AU145950	TGFB2		P*
AF116571	SOX13		P*
AI474666	IRS1		P
NM_016569	TBX3		P
NM_002309	LIF		P
NM_004503	HOXC6		P*
AI091372	CSRNP1		P

P positive correlation, *Direct target genes of Hedgehog signalings.

### Cell growth assays

Cell growth was determined using MTT [3-(4, 5 dimethyl-2-thiazolyl)-2.5-diphenyl- 2H-tetrazolium bromide] assays. Briefly, PC cell lines were plated in 96-well plates. MTT assays were performed after 12, 24, 48, and 72 hours and optical densities were determined at a wavelength of 490 nm.

### Liver metastases induction by splenic injection

Three groups of AsPC-1 cells (lentivirus-Gli1i, lentivirus-control, and lentivirus-SHH) were used to detect metastasis after intrasplenic inoculation into nude mice as previously described [9]. Briefly, mice were anesthetized with methoxyflurane, a minor abdominal left flank incision was made, and the spleen was exposed. AsPC-1 cells were injected into the spleen with a 30-gauge needle. The spleen was returned to the abdomen, and the wound was closed in one layer with wound clips. After 8 weeks, we harvested the liver and spleen and produced continuous frozen sections. We stained the sections with hematoxylin and eosin and counted spleen tumors, intrasplenic miniature metastases, and liver metastases under a fluorescence microscope and optical microscope. All animal experiment protocols used in this study were approved by the Animal Research Committee of Tongji University.

**Figure 5:**
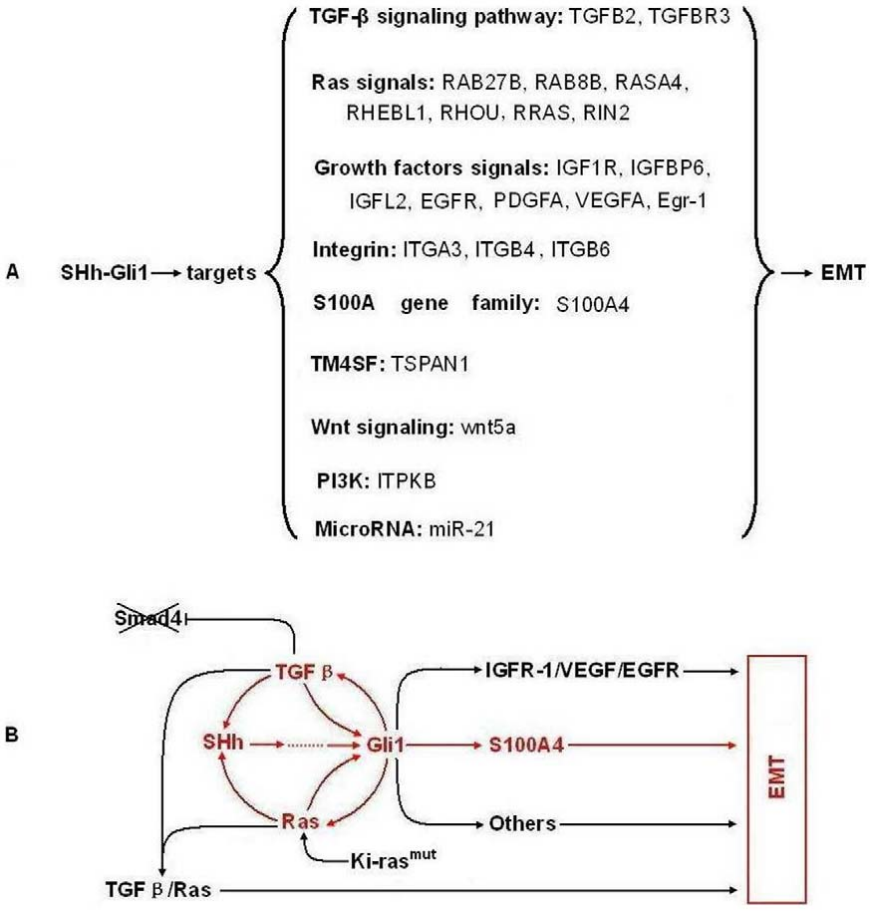
The EMT molecular network mediated by SHH-Gli1 signaling in PC cells. A: Target genes and signaling involved in EMT regulated by Gli1 in PC cells; B: The putative crosstalk model within the EMT molecular network mediated by SHH-Gli1 signaling.

### cDNA microarray analyses

AsPC-1 cells transduced with L-Gli1i and L-C were used in cDNA microarray assays with the Affymetrix Human Genome U133 Plus2.0 Array GeneChip. Three experiments were performed on a single total RNA preparation from the cells. Signal values are presented as the mean value of 3 replicate experiments. cDNA microarray assays and statistical analyses of the gene expression results were performed as described previously [10].

### Statistical Analyses

For all statistical analyses, we used SPSS17.0 software (SPSS, Inc, Chicago, IL, USA). Continuous variables are expressed as the mean ± SE. Non-paired Student's t-tests were used for statistical evaluation. *P*<0.05 was considered statistically significant.

## Results

### Lentiviral-Gli1i and -SHH transduction efficiency and PC cell EMT is regulated by SHH-Gli1 signaling

We transfected three PC cell lines with the lentiviral Gli1 interference vector (L-Gli1i), SHH over-expression vector (L-SHH), and control vector (L-C). We verified alterations in activation of SHH-Gli1 signaling by evaluating the expression of SHH, Gli1, and Patched1 using real-time RT-PCR. The real-time RT-PCR data revealed that the Gli1 and Patched1 genes were significantly downregulated by L-Gli1i transduction, whereas Gli1 and Patched1 were upregulated by L-SHH transduction compared with L-C (*P*<0.01; Figure 1A). Gli1 and Patched1 were target genes in most cell types with SHH signaling activated, therefore, the results suggest the lentiviral vectors efficiently changed the activation of SHH-Gli1 signals. The E-cadherin mRNA levels were drastically reduced by increased SHH/Gli1-expression in PC cells. A similar trend was observed with the E-cadherin protein.

### PC cell invasion and migration is regulated by SHH-Gli1 signaling

Data from the transwell assays showed that an increased number of cells from the PC cell lines invaded in a Gli1 dose-dependent manner through the Matrigel-coated filter within 24 hours (*P*<0.05; Figure 2A1–A9, B).

### The SHH-Gli1 signaling pathway regulates PC cell proliferation

Our MTT data showed that the L-Gli1i/SHH transduction did not significantly influence cell proliferation within 24 hours. However, after 48 hours, PC cell proliferation increased with viral transduction (Figure 2C1–C3).

### Liver metastases after injection of AsPC-1 cells into nude mice is regulated by SHH-Gli1 signaling

Our data from the nude mice model showed that 8 weeks after intrasplenic injection of AsPC-1 cells, there were spleen tumors in 8 of 10 mice in the L-Gli1i group, 8 of 9 mice in L-C group, and 9 of 9 animals in the L-SHH group. The average numbers of splenic miniature tumors were 2.6, 4.9, and 8.9, respectively. The incidence of liver metastases was 3 of 8 mice in the L-Gli1i group, 5 of 8 mice in the L-C group, and 8 of 9 animals in the L-SHH group. The average numbers of liver metastases were 2.7, 4.2, and 6.7, respectively (Figure 3, Table 2).

### cDNA microarray analyses of Gli1 target genes in AsPC-1 cells

The Patched1 gene, a direct target of Gli1, was upregulated 1.71341-fold in this study. Therefore, we set 1.7-fold regulation as the target gene standard. Using this threshold, the target gene profile data showed that 278 genes were upregulated and 59 genes were downregulated upon Gli1 in AsPC-1 cells. (Table 3). The regulated genes were classified into different categories based on well-documented and established biological or pathological function. Genes regulated by Gli1 belong principally belong to the following categories: cell invasion/migration, angiogenesis, cell survival, transport, metabolism, signal transduction, and immune system defense (Figure 4). We then compared these target genes with previous data by searching the Medline database to screen for differentially expressed PC genes and SHH signaling pathway target genes. Utilizing this approach, we identified 58 upregulated genes (Table 4) and 1 downregulated gene upon Gli1 inhibition in our screen that were previously been found to be similarly regulated in PC. Using the same method, we found 22 upregulated genes upon Gli1 inhibition that were previously found to be correlated with SHH signaling (Table 4). Moreover, 15 of 22 genes that were reported to be overexpressed in PC were involved in cell metastasis, including ITGB4, ANG, VEGFA, S100A4, WNT5A, and TGFB2 as well as cell survival, such as BCL2, BIRC3, IGFBP6, KLF4, and PLAU. At least 8 genes (WNT5A, BCL2, IGFBP6, PTCH1, MSX2, TGFB2, HOXC6, and SOX13) were previously demonstrated to be direct targets of SHH signaling [10], [11].

## Discussion

In this study, cell survival target genes could be divided into several types: (1) proliferation-related genes, such as IGFBP6, IGF1R, IRS1, EGFR, and ALOX5, (2) apoptosis-related genes, such as BIRC3 and Bcl-2, (3) Cell cycle-related genes, such as CCNG2, CDC2L6, and CDK6, and (4) CSC orCSCs maintenance-related genes. The stem cell phenotype predominantly included EMT, anti-treatment, and stem cell markers. The IGF signaling pathway was a key proliferation-related pathway and the Bcl-2 family was an important classic apoptotic signaling pathway. It was reported that Gli1 directly regulates CCND transcription and our data suggests it may regulate CCNG2 in the same manner [7]. The ABCC3 gene encodes multidrug resistance-associated protein 3 (MRP3), which is involved in chemotherapy resistance of cancer cells [12]. Moreover, MTS upregulation and CTPS downregulation has also been reported to lead to chemotherapy resistance [13]. In addition, KLF4 is a stem cell marker that promotes cancer stem cell population maintenance and CD59 upregulation may be associated with tumor cell immune escape [14], [15]. Interestingly, HUS1 downregulation likely weakens the DNA damage repair mechanisms [16].

Angiogenesis is necessary for cancer metastasis as well as for CSCs microenvironment maintenance. Substantial evidence suggests that activated SHH signaling may be one angiogenesis-initiating signaling pathway during pancreas carcinogenesis, though its exact mechanism is not known [17]. In this study, we found that Gli1 significantly upregulated pro-angiogenic factors, including ANG, VEGFA, PDGFA, TNFRSF12A, and IL-8, suggesting it has an important regulatory role in PC angiogenesis. Moreover, in this study, VEGF and PDGF were upregulated at the same time, suggesting that the proangiogenesis mechanisms of the SHH pathway are not just involved in endothelial cells (ECs) tuberformation, but also vessel wall maturation.

It was reported that SHH signaling pathway activation accompanied EMT, and EMT is required for migration of SHH-responsive cells during tissue morphogenesis. However, there was no evidence that Gli1 directly regulated Snail or Slug transcription. In the present study, target profile data showed that SHH signaling in EMT involved a complex crosstalk network (Figure 5A). The EMT-related target genes are summarized as follows: (1) TGF-β signaling pathway: *TGFβ2* and *TGFβR3*. Previous studies showed that TGFβ signaling is significantly elevated in PC with Smad4 mutation, resulting in the loss of Smad4-dependent cell growth inhibition and increased Smad4 independent EMT [18]. (2) Ras signaling pathway: RAB27B, RAB8B, RASA4, RHEBL1, RHOU, RRAS, and RIN2. Data indicate the Ras/ERK1/2 pathways are involved in the mesenchymal transformation of PC cells [19]. (3) Wnt signaling pathway: wnt5a. Previous study indicate that wnt5a promotes EMT through a non-classical pathway [20]. (4) PI3K/AKT signaling pathway: ITPKB. PI3K was found to strengthen Snail nuclear colonization through PAK1 activation of the AKT signaling pathway in EMT [21]. AKT functions as a central point to transduce extracellular (growth factors including insulin, IGF-1, and EGF) and intracellular (such as mutated or activated receptor tyrosine kinases, PTEN, Ras, and Src) signals [22]. (5) Growth factor and receptor signaling pathways: *IGF1R, IGFBP6, IGFL2, EGFR, PDGFA,* and *VEGFA.* Previous studies have demonstrated that abnormal activation of these pathways promotes epithelial-derived tumor expansion and progression through promotion of EMT-like transitions. Regarding mechanisms, IGFR signaling induces expression of the transcription factors Snail and Zeb [23]. PDGF may induce EMT via activation of the Wnt signaling pathway [24]. VEGF and EGF can increase of Snail and Twist protein expression [25]. (6) Integrins: I*TGA3, ITGB4,* and *ITGB6*. It has been reported that the α3 and β4 subunits can make up laminin-binding integrins with other subunits, such as α3β1 or α6β4, and these subunits can be palmitoylated that may contribute to integrin-tetraspanin interactions [26]. The potential prometastatic functions of these integrin subunits, particularly β4, were reported previously and tyrosine phosphorylation of the β4 Shc-binding site results in disassembly of hemidesmosomes and mobilization of signaling-activated α6β4 integrin. Mobilized α6β4 switches from keratin to actin filament association and may mediate migration and invasion of laminin isoforms [26]. (7) TM4SF: *TSPAN1.* TSPAN1 gene over-expression was detected in liver cancer, prostate cancer, gastric cancer, cervical cancer, and colorectal cancer [27]. It has been proposed that TSPAN1 gene expression correlates with cell proliferation and cancer prognosis. Our data suggests that TSPAN1, as a member of TM4SF, may participate in the EMT process of PC cells. However, it remains to be determined how it interacts with integrins, growth factors, or other TM4S proteins. (8) MicroRNAs: *miR-21*. Studies have shown that miR-21 is associated with PC metastasis and prognosis and may play a role in TGF-β -induced EMT [28], [29]. (9) S100A gene family: *S100A4*. It has been reported that S100A4 and E-cadherin are inversely regulated in several cell systems and that S100A4 promotes the expression of the essential transcription factors, Twist and Snail, in the EMT process, as well as mesenchymal markers, including vimentin and MMPs [30], [31].

Interesting, our data suggests that the EMT molecular network mediated by SHH signaling may contain at least two important positive feedback loops in PC cells. The first is the positive feedback between SHH and TGFβ signaling. *In vitro* and *in vivo* evidence suggests the crosstalk between TGFβ and SHH results in reciprocal induction. TGFβ upregulated SHH and activated Gli1 during EMT induction; however, SHH signaling upregulated TGFβ2 and TGFBR3 as demonstrated in this and a previous study [32], [33]. The second positive feedback loop is between SHH and Ras signaling. Previously, studies showed that k-ras mutation was an essential mechanism of SHH and Gli1 upregulation in PC cells and in this study, we found that Gli1 upregulated several Ras-related genes to activate Ras signaling [34].

Based on previous studies and our data, we speculate that this molecular network might start with *k-ras* mutations, followed by SHH signaling activation, and finally, the TGFβ signal joins and a positive feedback loop forms between the three pathways. The SHH signal was continuously enhanced through this positive feedback and directly promotes EMT via regulation of EMT-related Gli1 target genes, such as *IGFR1, VEGF, EGF,* and *S100A4*. (Figure 5B). However, this molecular network model may be more complex with the participation of additional signaling proteins, such as integrins, PI3K/AKT, and WNT.
